# Supplementary material for: Asymmetric Synthesis of Quaternary Hydantoins via a Palladium-Catalyzed Aza-Heck Cyclization
Source: J Am Chem Soc. 2025 Nov 14;147(49):44692–8. doi: 10.1021/jacs.5c16022 (PMC12703750; doi:10.1021/jacs.5c16022)
Supplement: Supplementary file 2 [file ja5c16022_si_002.zip › All NMR FID Files/5/5_CarbonNMR/3/pdata/1/email_Model-Hyd-13C_3_1.pdf]

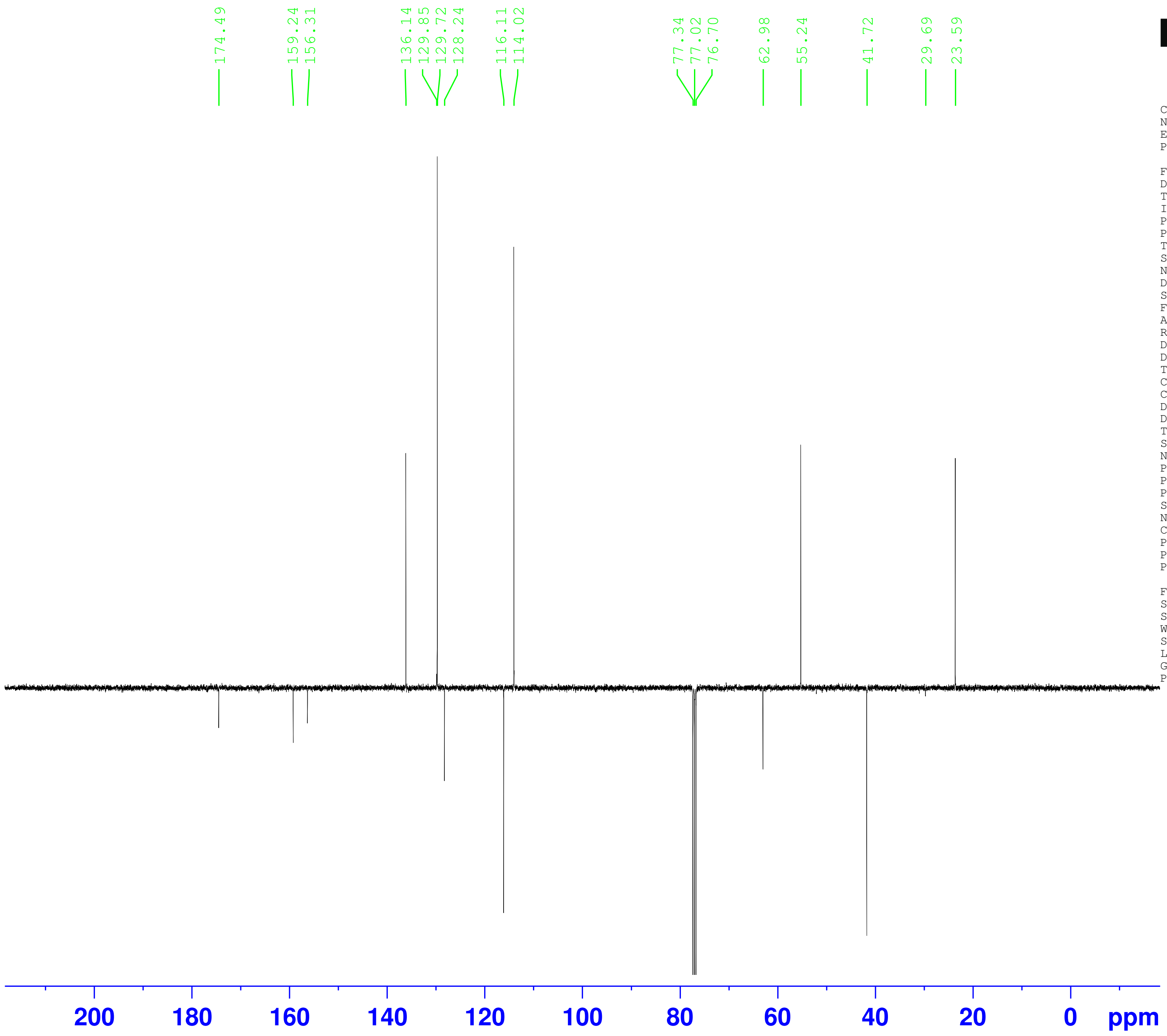


```
Current Data Parameters
NAME       Model-Hyd-13C
EXPNO                  3
PROCNO                 1

F2 - Acquisition Parameters
Date_           20250628
Time               21.45 h
INSTRUM   Avance Neo 400 U of DE Brown Lab
PROBHD    Z104450_0412 (
PULPROG             jmod
TD                 65536
SOLVENT            CDCl3
NS                  1024
DS                     4
SWH            23809.524 Hz
FIDRES          0.726609 Hz
AQ             1.3762560 sec
RG                   101
DW                21.000 usec
DE                  6.50 usec
TE                 300.0 K
CNST2        145.0000000
CNST11         1.0000000
D1            5.00000000 sec
D20           0.00689655 sec
TD0                    1
SFO1         100.6228298 MHz
NUC1                 13C
P1                 10.00 usec
P2                 20.00 usec
PLW1         44.93500137 W
SFO2         400.1316005 MHz
NUC2                  1H
CPDPRG[2         waltz65
PCPD2              90.00 usec
PLW2          8.31639957 W
PLW12         0.23101000 W

F2 - Processing parameters
SI                 32768
SF           100.6127685 MHz
WDW                   EM
SSB                    0
LB                  1.00 Hz
GB                     0
PC                  1.40
```
